# An Assessment of Vape Shop Products in California before and after Implementation of FDA and State Regulations

**DOI:** 10.3390/ijerph192315827

**Published:** 2022-11-28

**Authors:** Ellen Galstyan, Artur Galimov, Leah Meza, Jimi Huh, Carla J. Berg, Jennifer B. Unger, Lourdes Baezconde-Garbanati, Steve Sussman

**Affiliations:** 1 Institute for Health Promotion and Disease Prevention Research, Department of Population and Public Health Sciences, Keck School of Medicine, University of Southern California, Los Angeles, CA 90007, USA; 2 Department of Prevention and Community Health, Milken Institute School of Public Health, GW Cancer Center, George Washington University, Science & Engineering Hall, 800 22nd St. NW, #7000C, Washington, DC 20052, USA; 3 Department of Psychology, University of Southern California, Los Angeles, CA 90007, USA; 4 School of Social Work, University of Southern California, Los Angeles, CA 90007, USA

**Keywords:** e-cigarettes, ENDS, nicotine, vape shops, e-cigarette retailers, U.S. Food and Drug Administration, Deeming Rule

## Abstract

Vape shops specialize in sales of e-cigarettes and related products. This study examines whether vape shops adapted their products and services in response to changes in federal and state policies that affect the tobacco retail environment between 2014–2022. In this multicohort study, four waves of study data were used to examine the trends in products sold in vape shops in Southern California. Items sold were assessed through systematic store product observations and included categories of e-cigarettes, device modification equipment, and other products (e.g., Cannabidiol (CBD), paraphernalia). Descriptive statistics are reported. The availability of disposable devices increased from 18% at Wave 1 to 98% of shops at Wave 4. Pod mods were first observed in 79% of the shops beginning at Wave 3. Device modification drills later become obsolete, from 60% at Wave 1 to 0 by Wave 4; self-service sampling displays declined from 83% of shops to 9%. Vape shops did not carry CBD products until Wave 3 (2017/2018), when 19.0% of shops carried CBD products and 72.9% at Wave 4. Future research should examine how e-cigarette retailers and manufacturers respond to changing state and federal regulations to better understand the implications of regulatory efforts.

## 1. Introduction

Vape shops and e-cigarette companies play a critical role in shaping the market and affecting the distribution and marketing of e-cigarettes and related products [1]. Since e-cigarettes entered the U.S. market in 2006–2007, their distribution and purchase channels have greatly evolved [1,2]. Vape shops and the e-cigarette industry have gone through constant changes to meet consumer demand in a competitive industry while facing changing state and federal policies and regulations [3]. These shops differentiate themselves from other tobacco outlets in that vape shop retailers offer a wider selection of “alternative nicotine products” and claim to “promote healthy lifestyle changes for individuals that want to quit smoking combustible cigarettes by switching to vaping products” [4,5]. However, these retailers also provide unsubstantiated claims regarding e-cigarettes’ safety, health benefits, [6] and utility for cigarette cessation, as there is conflicting evidence about whether e-cigarettes are an effective cessation device [7,8].

### 1.1. Federal Regulations

#### 1.1.1. The United States Food and Drug Administration (FDA) Deeming Rule 

Vape shops and e-cigarette use remained largely unregulated up until 2016, when the U.S. Food and Drug Administration (FDA) passed the federal “Deeming Rule”, extending its regulatory authority to e-cigarettes, effective 8 August 2016 [9]. This gave the FDA authority to monitor and regulate the marketing, manufacturing, and distribution of e-cigarettes and related products [10,11]. This set the stage for regulatory action to enforce: (1) required health warning labels on products (2) monitor health claims made by manufacturers and retailers and prohibiting misleading advertising (3) prohibit free sampling/self-service and e-cigarette device modification, unless licensed as a manufacturer (i.e., prohibit shops from using electric drills for modification of device atomizers, coils, mouth tips or batteries), (4) required submission of premarket review of tobacco products and disclosure of information about ingredients, product design, health risks, and product appeal to youth from manufacturers and (5) intercede on e-cigarette sales to those underage (i.e., 18 years old from 2016 to 2020, 21 years old from 2020 to present) [9,10]. 

#### 1.1.2. The E-Cigarette Flavor Ban 

In January 2020, the FDA issued an enforcement policy against companies that do not cease manufacturing, distribution, and sales of unauthorized flavored cartridge-based e-cigarettes other than tobacco or menthol flavors [9,10]. Many flavored e-cigarettes remain on the market due to loopholes in federal policy, which only limits flavors for “closed pod” e-cigarettes, and exempts other types, including disposable pod and open-system, refillable devices [12]. To avoid additional FDA oversight, e-cigarette manufacturers discovered another loophole and began to utilize synthetic nicotine in devices, marketed as “tobacco-free nicotine” or nicotine not derived from tobacco plants (which would not fall under regulatory authority). Manufactures of synthetic nicotine products claim they are an important advance, because tobacco leaf-derived nicotine may contain some contaminants, including carcinogenic ones, whereas synthetic nicotine is a “pure” form of nicotine [13]. This product is chemically identical to the nicotine from tobacco plants, and similarly addictive, but only recently falls within the FDA’s tobacco-related authority [13,14]. Recent sales data show that since the FDA increased e-cigarette enforcement in February 2020, sales of disposable pod fruit- and candy-flavored devices have grown by 290%, to 6.46 million devices a month by November 2021 [13]. It is still unclear whether synthetic nicotine products contain contaminants or otherwise have the same harmful effects as nicotine derived from tobacco plants.

#### 1.1.3. The U.S. Preventing All Cigarette Trafficking (PACT) Act 

The Preventing All Cigarette Trafficking Act (PACT Act) was created by Congress in 2010 and authorizes The Bureau of Alcohol, Tobacco, Firearms and Explosives (ATF) to prevent organizations from profiting from the illegal sales of tobacco products and imposes penalties for avoiding sales tax payments [15]. On 27 March 2021, Congress extended the (PACT) Act to include e-cigarettes and prohibits United States Postal Service (USPS) delivery of cigarettes and smokeless tobacco to consumers. Tobacco sellers are required by federal, state, and local laws to meet sales reporting, tobacco product shipping and tax requirements to ensure they are compliant with cigarette regulations [16]. This new law may drive more consumers to vape shops, many of which have continued to sell flavored and unregulated e-cigarette products, largely in part to gaps in FDA enforcement policies and non-standard compliance checks [17,18].

### 1.2. California State Regulations

#### 1.2.1. California Proposition 64: The Adult Use of Marijuana Act 

**Figure 1:**
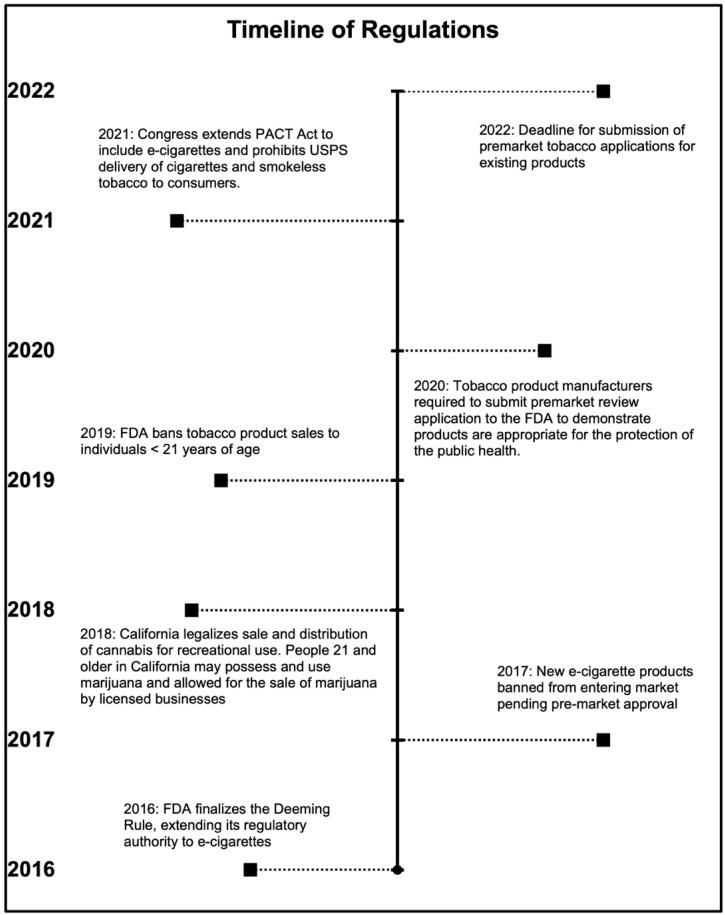
Timeline of Relevant State and FDA Regulations.

As of January 2018, California legalized the sale and distribution of cannabis in both a dry and concentrated form for recreational use [19,20]. This made it legal for people 21 and older in California to possess and use marijuana and allowed for the sale of marijuana by businesses licensed to do so. While shops licensed as tobacco retailers are prohibited from carrying marijuana products in California, this has broadened the range of products sold at vape shops, as some open system devices can be used to vape marijuana and cannabis oil [21]. More commonly found in vape shops now are tobacco pipes or wrapping papers (which are widely used to smoke marijuana), Delta-8-tetrahydrocannabinol and other cannabidiol products, such as CBD oil, e-juice, candies/gummies, tinctures, lip balms, and salves (tobacco retailers can only sell products that contain ≤0.3% of THC). While US states that permit recreational cannabis use require products to be sold by licensed dispensaries, many vape shops sell THC derivatives (such as delta-8-THC, delta-10-THC, delta-HHC-THC, or delta-O-THC) and pre-mixed products that resemble cannabis products. Among the major concerns regarding these unregulated, psychoactive products are their contents and the inaccurate THC derivative labeling, which has significant implications for consumer safety [22,23,24] [See Figure 1 for tobacco regulations].

#### 1.2.2. E-Cigarette Products

Among the most critical drivers of e-cigarette consumerism is the variety of flavors, devices, and accessories. The availability of flavors in e-cigarettes is a key reason for e-cigarette use among former and current adult cigarette users as well as youth who may perceive flavored e-cigarettes as less harmful than traditional cigarettes [25,26]. Many vape shops offered a social atmosphere with customized e-liquids mixed on site, samples available for consumers to try while inside the store, and vaping competitions (“cloud chasing”) [11]. Moreover, vaping devices sold at vape shops have evolved from thin disposable devices resembling the shape of a cigarette (1st generation), to tubular vape pens with a fillable tank (2nd generation), to box-shaped mod devices (3rd generation), to pod mods and disposable pods (4th generation) [27,28]. Disposable pod devices advertise anywhere between 3000 to 5000 “puffs” and contain salt-based nicotine liquid (some may contain 20–70 mg/mL of nicotine) [27,28].

To address the gaps in literature examining vape shop products and the effects of regulations on what is sold in these shops, the present study provides an examination of the breadth of products in Los Angeles vape shops from 2014 to 2022 during changing regulations. The constant change of e-cigarette products, regulations and unstandardized compliance checks highlight the importance of obtaining data on how vape shops respond to changes in policies. The present study fills an important research gap by exploring the changes in e-cigarette product availability over the course of 8 years during regulatory change. We hypothesized that vape shops will: (1) increasingly offer devices that meet (bypass) regulatory standards, even if they may be more harmful, such as disposables pod devices with high levels of nicotine and synthetic nicotine, (2) diversify by increasingly selling CBD and marijuana-related products, (3) show reductions in the availability of device modification services and products, including mouth tips, wires, coils, and drills (which would need FDA approval as a manufacturer), (4) show fewer self-sampling displays since free sampling of products are no longer permitted and (5) show increases in unregulated e-cigarette devices (e-cigarette products that do not have FDA pre-market approval). This study may help to inform regulatory authorities of the prevalence of these products in vape shops, the indirect effects lax regulations may have on products that are marketed in shops (regulation loopholes) and the potential causes of increasing rates of e-cigarette smoking initiation among youth and young adults.

## 2. Materials and Methods

### 2.1. Initial Sample Recruitment 

We recruited Southern California vape shops in neighborhoods in San Bernardino, Orange, Riverside, and Los Angeles Counties [11]. 

Vape shops that sold exclusively e-cigarette devices, products, and juices (“vape-only” shops) were included in the 2014 sample (N = 77). Smoke shops and vape/smoke shops that sold a majority of combustible tobacco products were not included in the study. Selecting vape shops for the sample was achieved by using Google Reviews, Yelp reviews and photos, and shop websites [4,7,8,11]. To increase the sample size and replace shops that had gone out of business at Waves 1 and 2, 84 additional vape shops in cities where data had been collected previously were added to the Wave 3 sample using Yelp and Google searches.

**Figure 2:**
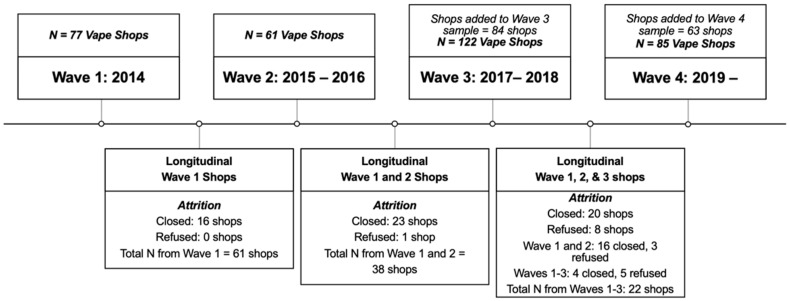
Data Collection Timeline.

The current study took place between 2014–2022. Baseline data collection took place from June 19, 2014, to December 6, 2014 (Wave 1). Employees at 77 shops provided consent to participate in our study. The first follow-up (Wave 2) of all open shops from baseline took place from July 2015 to January 2016 (N = 61). Then, 2-1/2 years post-baseline data collection (Wave 3: 17 November 2017–11 December 2018) of all open shops followed up from Wave 2 (N = 38) and an additional cohort of 84 shops were assessed (total N = 122). During Wave 4 of the study, 27 of the previous shops from Wave 3 closed and 63 additional shops were added to the sample. Wave 4 of data collection started in July 2019 and is currently ongoing. Wave 4 currently includes 85 vape shops in Southern California, 22 of which were from the original Wave 1 shops (see Figure 2).

### 2.2. Data Collection across the Four Waves

Groups of two or three team members visited each of the vape shops for recruitment and consent to collect data between the hours of 10 am and 6 pm on weekdays and some weekends. All Internal Review Board (IRB) protocols were followed, and employees and owners were assured that the information collected would be anonymous. After obtaining verbal consent, team members conducted the data collection, which took about 35 min to complete, and included recording shop characteristics with a shop observation checklist using an adapted version of the Vape Shop Standardized Tobacco Assessment for Retail Settings surveillance tool (V-STARS) [29]. Shop employees or owners who agreed to participate in the study were offered a $50 gift card. 

### 2.3. Measures

Presence of products of interest in the shop product measure were coded (1 = yes, 0 = no) with a three-page checklist. The store observation checklist included assessments of availability (yes/no) for: (1) categories of e-cigarettes or other electronic devices and related products (e.g., disposable e-pens, atomizers, pod-mod devices, mouth tips, wires/coils; a pod-mod device category was added at Wave 3 to capture this category as it emerged); (2) Cannabinoid products (e.g., gummies, drops, e-juice, lip balms; added after Wave 3 as these products emerged in vape shops) and other drug-related items (e.g., glass pipes, bongs); and (3) customer amenities, specifically drills—which allow device modification—and self-service e-juice sampling displays. 

### 2.4. Data Analysis

Descriptive statistics were used to examine the trends of products sold at shops and availability of customer amenities at each shop during each time point and across the four waves of data collection. Inter-rater reliability checks on presence of key items (e.g., disposables, box mods, pod mods, CBD, drills, and self-service displays) revealed high inter-rater agreement (all Kappa Coefficients > 0.60) [30].

## 3. Results

### 3.1. E-Cigarette Products

Among 77 vape shops assessed at Wave 1 (baseline; 2014), 14 (18.2%) sold disposable vaping devices, 76 (98.7%) sold both atomizers and mouth tips, and 10 (13.0%) sold wires/coils for device modification. Among the 61 shops assessed at Wave 2 (2015), 6 (9.8%) sold disposable vaping devices, 60 (98.4%) sold atomizers, and 40 (65.6%) sold wires/coils for device modification. At Wave 1 and 2, pod mod devices were not yet on the market (they were invented in 2015). Among the 122 shops in Wave 3 (2017), 11 (9.0%) sold disposable vaping devices, 119 (97.5%) sold atomizers, 97 shops (79.5%) sold some type of pod mod device, and 90 (73.8%) sold wires/coils for device modification. Among the 85 shops assessed at Wave 4 (2019–2022), 84 shops (98.8%) sold disposable vaping devices, 77 (90.5%) sold atomizers, 82 shops (96.5%) sold some type of pod mod device, and 44 (51.8%) sold wires/coils for device modification.

### 3.2. Customer Amenities 

After federal regulations barred vape shop employees from making device modifications for customers, the availability of drills in shops that were used for device modification decreased. In 2014, 46 of 77 (60%) observed shops had some type of drill or workstation for product modification. In Wave 2, 37 of 61 (60.7%) shops had drills and in Wave 3 only 20 of 122 (16.4%) shops still had drills. In Wave 4, no shops were observed with a drill present. 

At Wave 1, 64 of 77 (83.1%) shops had free sampling or some type of sample e-juice station set up for customers to smell and try e-juices before purchase. At Wave 2, 54 of 61 shops (88.5%) had free sampling, and at Wave 3, after free sampling was prohibited, 13 (10.7%) shops had a sampling station. At Wave 4, only 8 of 85 (9.0%) shops had e-juice self-service sampling displays.

### 3.3. Other Products 

In 2018 during Wave 3 (after the legalization of marijuana in California), vape shops started to carry marijuana-related paraphernalia. In Wave 3, 26 out of 122 shops (21.0%) carried some type of glass pipe, “bong”, or drug-related product. At Wave 4, 26 of 85 assessed shops (30.5%) had these products. 

At Wave 1 and Wave 2, no shops in this sample sold CBD products. However, at Wave 3, 23 of 122 (19.0%) shops carried CBD products, and at Wave 4, 62 of 85 (72.9%) shops carried CBD. More recently during Wave 4, shops have started to carry Delta-8- and Delta-9 products, this was observed in 9 of 85 (10.6%) shops [See Table 1].

## 4. Discussion

This study provides novel findings documenting the change of products sold in vape shops in Southern California over a span of 8 years throughout the implementation of federal and state regulations. In 2016, federal regulations banned the modification of vape devices in shops, requiring a manufacturer’s license to do so [9,10]. This may explain why vape shops slowly decreased sales of wires and coils from year to year, eliminated drills available in shops by 2020, and moved toward easy to use disposable and pod mod devices. After free e-juice samples became prohibited federally, the number of self-service displays decreased dramatically.

In December 2019, the Food and Drug Administration (FDA) announced that it will enforce stricter e-cigarette regulations, including flavor bans (on flavors other than tobacco, mint, and menthol) and a mandatory submission of a pre-market tobacco application for flavored cartridge-based products sold at vape shops [9,10]. In January 2020, the US Food and Drug Administration (FDA) issued an enforcement policy against companies that do not cease manufacture, distribution, and sales of unauthorized flavored cartridge-based e-cigarettes other than tobacco or menthol flavors.

Historically, the e-cigarette industry has differentiated itself from the tobacco industry by marketing and promoting e-cigarette use as a less harmful alternative to combustible cigarette smoking and by selling vaping devices that provide opportunities for smokers to quit [4,31,32]. Vape shops may play a key role in providing customers with product recommendations to suit each individual’s needs (including toward potential goals of combustible cigarette cessation) and adapt to current vaping-related regulatory information. Conversely, due to the need to diversify, several of the surviving shops from among the longitudinal shops began marketing heavily combustible cigarettes. For users who historically accessed e-cigarettes at vape shops, stores that transitioned to selling other tobacco products provide a greater exposure to the marketing and availability of such products. Prior research indicates that the exposure to tobacco products, marketing, and retail undermines smoking cessation efforts [33,34,35]. Given that many people use e-cigarettes to try to quit smoking cigarettes or maintain abstinence from cigarettes, this is a major concern [34,36,37].

Because of current policies, some vape shops may go out of business or begin selling other tobacco products and convert to smoke shops; moreover, it is possible that vape shop customers trying to quit conventional cigarettes may return to using combustible cigarettes, find ways to make their own products or purchase unregulated products online, which arguably could negatively impact public health efforts [37,38].

The factors that impact the products sold and marketed at vape shops may be a direct reflection of FDA, state, and local policies (e.g., free sample prohibition and self-service display presence). Regulations that are implemented may not be as closely monitored by agencies. These data have shown that shops sell market products that, although fall under strict regulatory guidelines, may be more harmful (e.g., synthetic nicotine, salt-based devices). This highlights the need for broader regulatory definitions and active surveillance and enforcement of regulations on e-cigarette product sales.

### Limitations 

There are several study limitations to consider. The sample of vape shops were in Southern California, which might limit generalizability to other geographic areas. To enhance generalizability, future research should compare observations across different U.S. regions, and perhaps, different countries. Additionally, there was an overlap, but also a nonoverlap, of shops at later waves of collection as some shops closed. Still, a sufficient number of shops were assessed at each wave, and changes in product prevalence in shops were large enough such that one may infer changes were due to changes in products and not differences in shops. This study did not collect detailed data regarding shop employees’ or owners’ opinions on what direct effects the current regulations had on their shops, when new products started to be marketed and sold, length of product shelf life, or volume and structure of sales of these products. In addition to these limitations, the V-STARS for point-of-sale assessment is often limited and the collection of sales volume data is considered proprietary information and difficult to track in the vape shop industry [39,40]. Methodologically, this introduces a challenge for similar endeavors as new categories of items continue to be rapidly introduced to the market in documenting longitudinal change in the landscape of e-cigarette products. This study was halted in March 2020 until November 2021 due to the COVID-19 Pandemic, which may have caused many shops to close. 

## 5. Conclusions

Growing rates of vaping young adults, and non-smokers, and problems associated with youth access [41,42,43] have resulted in more urgency by the public and more pressure for policymakers to implement broader regulatory changes [24,26,27]. Regulations imposed on these shops and the industry have been effective; however, new products that may be potentially more harmful but manufactured to meet new regulation standards have emerged and may lead to unintended health effects. Results from this study add to existing data and highlight how e-cigarette and marijuana-related products sold and marketed in vape shops have evolved and changed because of policy loopholes and broad definitions in regulations. There is a need for increased tracking of novel nicotine-containing products (e.g., oral nicotine products, such as pouches and gum), nicotine-juice concentrates that contain high levels of nicotine, as well as monitoring of marijuana-related products sold at these shops. Implications for future federal regulations of different variations of nicotine products, closer monitoring of unregulated marijuana products sold to the public and surveillance efforts that address loopholes in current and future regulations are needed.

## Figures and Tables

**Table 1:** Products Sold in Vape Shops by Year (A non-longitudinal sample of retailers).

	Wave 1 (N = 77)	Wave 2 * (N = 61)	Wave 3 * (N = 122)	Wave 4 ** (N = 85)
***E-cigarette Products***				
Disposable Devices	14 (18.2%)	6 (9.8%)	11 (9.0%)	84 (98.8%)
Atomizers	76 (98.7%)	60 (98.4%)	119 (97.5%)	77 (90.5%)
Mouth tips	76 (98.7%)	60 (98.4%)	111 (91.0%)	N/A
Pod Mod Devices	N/A	N/A	97 (79.5%)	82 (96.5%)
Wires/Coils	10 (13.0%)	40 (65.6%)	90 (73.8%)	44 (51.8%)
***Customer Amenities***				
Self-Service E-juice Display	64 (83.1%)	54 (88.5%)	13 (10.7%)	8 (9.0%)
Drills Available in Shop	46 (60.0%)	37 (60.7%)	20 (16.4%)	0
***Other Products***				
Glass pipes, bongs, etc.	0	0	26 (21.0%)	26 (30.5%)
CBD	0	0	23 (19.0%)	62 (72.9%)
Delta 8/9-THC	0	0	0	9 (10.6%)

* Wave 2 data is a subset of all shops that were still open from Wave 1. Waves 3 and 4 include shops that were still open from Wave 1 as well as “replacement shops” for shops that have closed. ** Note: 120 shops will be assessed in Wave 4 (data collection is still ongoing).

## Data Availability

Not applicable.
